# Alfalfa/Lucerne (*Medicago sativa*) as a Source of Functional Bioadditives for Elastomeric Natural Rubber Composites

**DOI:** 10.3390/polym16233444

**Published:** 2024-12-09

**Authors:** Justyna Miedzianowska-Masłowska, Marcin Masłowski, Krzysztof Strzelec

**Affiliations:** Institute of Polymer and Dye Technology, Lodz University of Technology, Stefanowskiego 16, 90-537 Lodz, Poland

**Keywords:** biocomposites, natural rubber, Alfalfa, biomass, phyto-ashes, freeze-dried natural extracts, functional properties

## Abstract

This study explores the impact of three bioadditives derived from Alfalfa—biomass, bio-ashes, and lyophilisates—on natural rubber composites, with a primary focus on the anti-aging properties of lyophilisates. Composite samples were prepared by incorporating these bioadditives into natural rubber and analyzed using various characterization techniques to evaluate mechanical, thermal, aging, and surface properties. The results highlight the promising anti-aging effects of lyophilisates, significantly enhancing the aging resistance of natural rubber. The aging factor was the closest to unity among all systems. Biomass and bio-ashes were also examined, offering insights into their influence on tensile strength, viscoelasticity, and surface wettability. The tensile strength values were almost 50% higher than those of the reference sample (8.5 MPa). The study provides a detailed understanding of the interactions between these bioadditives and natural rubber, showcasing their potential to enhance elastomer performance. These findings underscore the viability of Alfalfa-based bioadditives as sustainable options for improving rubber properties, with significant implications for industrial applications.

## 1. Introduction

Composite materials that incorporate one or more phases derived from biological sources are called biocomposites. Biocomposites often involve plant fibers, such as those obtained from wastepaper, recycled wood, flax, hemp, cotton, and agricultural crop residues. These materials present numerous benefits, such as their renewable nature, lightweight composition, biodegradability, cost-efficiency, and exceptional specific strength [1,2]. The interest in developing both biodegradable and non-biodegradable bio-based polymer composites has recently increased due to the need to find an alternative to fossil-based polymers.

Non-biodegradable elastomeric biocomposites are advanced materials that combine elastomers with biodegradable components, resulting in unique properties and improved environmental sustainability. These composites offer an alternative to conventional nonbiodegradable elastomers, which pose significant challenges in terms of their disposal and long-term environmental impact [3]. By incorporating biodegradable fillers or reinforcing agents into elastomeric matrices, nonbiodegradable elastomeric biocomposites provide enhanced mechanical properties, such as improved tensile strength, modulus, and elongation at break. Additionally, they exhibit desirable characteristics such as biocompatibility, thermal stability, and resistance to aging. These biocomposites can find applications in a wide range of industries, including automotive, aerospace, packaging, and biomedical industries, where the demand for sustainable materials is rapidly increasing [4]. The development and utilization of nonbiodegradable elastomeric biocomposites contribute to the advancement of eco-friendly materials, addressing the global need for sustainable solutions in various sectors while maintaining the desirable properties of elastomeric materials.

Alfalfa (*Medicago sativa*), a perennial plant in the legume family (Fabaceae), originates from the Mediterranean but is now cultivated worldwide. It serves as a nutrient-rich livestock feed and has a history of use in herbal medicine, with reputed benefits as a diuretic, digestive aid, and source of antioxidants [5,6,7]. In some cultures, alfalfa is also employed to address arthritis and hormonal imbalances. The plant’s sprouts are widely consumed in salads and sandwiches. Alfalfa’s nutritional and medicinal properties stem from its diverse phytochemicals, including flavonoids, coumarins, saponins, alkaloids, and phenolic acids, which exhibit antioxidant, anti-inflammatory, and antimicrobial activities [8,9]. Rich in vitamins (A, C, E, and K) and essential minerals such as potassium, iron, calcium, manganese, silicon, and phosphorus, alfalfa represents a valuable dietary and therapeutic resource [10,11].

*Medicago sativa* can be considered as an addition to elastomeric mixtures, offering several technological and product benefits. The high content of cellulose and lignin in its fibers allows alfalfa to act as a natural filler, providing reinforcement and improving the mechanical properties of rubber composites. It is also a renewable raw material that, compared to synthetic fillers such as carbon black or silica, can serve as a cost-effective alternative, further promoting sustainable development. Silica and carbon black, the most common rubber fillers, are currently more cost-effective due to large-scale production. However, plant-based bioadditives, derived from renewable and highly abundant plant resources, are gaining ground in sustainability and could become competitive with advancements in technology and growing demand for eco-friendly solutions. Future profitability may rise with improved production methods, increasing costs of conventional fillers due to regulations, and higher demand for sustainable products. However, it is very difficult to find information in the literature on the use of alfalfa as a bioadditive for various types of elastomeric materials, including natural rubber. Therefore, this work will aim to check the impact of the addition of alfalfa in various forms, such as biomass, phyto-ash, or freeze-dried natural extracts, on the chemical and physical properties of vulcanizates.

The use of bioextracts in elastomer technology can offer a number of benefits and opportunities. Antioxidant-rich bioextracts can be used as natural alternatives to synthetic antioxidants in rubber compounds. These antioxidants help protect rubber from thermo-oxidative degradation, extending its life and improving its performance in various applications. Furthermore, some bioextractives, such as cellulose fibers derived from plant sources, can act as strengthening or processing agents (plasticizers) in rubber compounds [12]. These natural fibers can improve the mechanical and processing properties of the rubber compound [13,14,15].

Phyto-ash, in turn, is a natural alternative to mineral fillers such as silica or calcium carbonate. The addition of phyto-ashes, which has already been proven by the authors of [16], can improve the mechanical properties of rubber, such as tensile strength, modulus, and tear resistance, improving the performance of rubber products [17,18]. Some phyto-ashes, especially those from plants with high mineral content, may have flame-retardant properties. Adding these ashes to rubber compounds can improve the fire resistance of rubber materials, making them suitable for applications where fire safety is critical [19]. Adding phyto-ashes can improve the thermal conductivity of rubber compounds, preventing heat build-up and improving overall performance. Phyto-ash may contain alkaline compounds that can be used to regulate pH in rubber formulations. Balancing pH levels is essential to maintaining the stability and properties of rubber compounds during processing and curing.

The use of both plant biomass, phyto-ashes, and plant extracts in the natural rubber industry promotes sustainable practices and waste disposal. Bio-based additives can be derived from agricultural residues or by-products, reducing waste and providing an environmentally friendly alternative to synthetic fillers. When using them, it is important to consider factors such as the type of plant material used in production, composition, particle size, and the specific application requirements of the rubber. As is evident from the presented literature review, further research and development are required to optimize the properties and compatibility of natural bioadditives with rubber materials, ensuring their effectiveness and suitability for various rubber applications, which is the main concept of the research presented in this article.

The scientific novelty of this work lies in the development of alfalfa/lucerne (*Medicago sativa*) for the production of bioadditives in elastomeric compositions. This is achieved by exploring various methods of processing plant material to obtain functional agents that impart specific properties to polymer materials. The study presents a comprehensive, multi-faceted approach to harnessing the potential of plants in elastomer technology. Typically, research on polymer biocomposites focuses on determining the effect of adding natural fibers on various properties. However, the work introduces a completely different concept, emphasizing the full utilization of plant material, which, after appropriate processing, serves as functional additives. The article demonstrates that each selected bioadditive (biomass, phyto-ashes, and extracts) from alfalfa uniquely influences the properties of rubber, including its spatial structure, mechanical, thermal, barrier, and anti-aging characteristics. Depending on the desired effect, different types of additives derived from natural sources can be employed, or combinations of these additives can be used to enhance multiple functional properties simultaneously. Moreover, the use of plant materials to produce bioadditives promotes the development of new technologies based on renewable raw materials. This work offers new insights into bio-based products that could potentially replace synthetic and fossil-based fillers or auxiliary agents, such as silica, carbon black, or anti-aging substances, in the future.

## 2. Materials and Methods

**Research objects**

- Raw materials for obtaining bioadditives:

Alfalfa *(Medicago sativa)*—dried plant, provided by Manutea (Chalupki, Polska).

- Elastomer:

Natural rubber in the form of Ribbed Smoked Sheet (RSS I), delivered by Torimex Chemicals (Lodz, Poland).

- Conventional cross-linking system:

Sulfur (S)—cross-linking agent; 2-mercaptobenzothiazole (MBT)—vulcanization accelerator;

Zinc oxide (ZnO)—cross-linking activator;

Stearic acid (SA).

All components were provided by Merck Life Science (Darmstadt, Niemcy)

**Preparation of biomass (BM)**

The dried alfalfa herb was ground in a ball mill (8000D, SPEX) with a mechanism of vibrating vessel movements, during which the inertial displacement of the balls and the sample relative to the vessel walls caused crushing and grinding of the material. The process of grinding one batch (approx. 2 × 10 g) lasted 30 min.

**Preparation of lyophilisates (LP)**

First, extracts of the alfalfa herb were prepared using an extractor (Ser 148, VELP). Cellulose thimbles containing 15 g of plant powder were extracted in 100 mL of a mixture of water and ethanol (50:50; *v:v*) for 2 h. In order to reduce the amount of solvent, the extracts were subjected to evaporation (80 °C, 250 mbar). After this stage, the frozen extract was transferred to a lyophilizer (FreeZone 2.5 Plus Freeze Dryer, Labconco, Kansas City, MO, USA), and the lyophilization process was carried out for 48 h under reduced pressure and a temperature of −85 °C.

**Preparation of phyto-ashes (PAs)**

To obtain phyto-ash, 50 g of alfalfa powder was placed in a ceramic crucible. The material was then heated in a muffle furnace at a rate of 10 °C per minute until the maximum temperature of 600 °C was reached. The combustion process lasted approximately 2 h. This process was performed multiple times until the desired quantity of phyto-ashes was obtained.

**Preparation of elastomeric composites**

The process commenced by introducing the selected additives, as mentioned previously, into the natural rubber. Initially, the mixing apparatus (Measuring Mixer 30/50, Brabender, Graz, Austria) was employed to plasticize the natural rubber using rotors set at a temperature of 50 °C and a rotation speed of 45 rpm for approximately 4 min. Subsequently, the additives were incorporated into the mixture, and the blending process for each sample continued for a duration of up to 15 min.

Following the completion of the blending step, the samples containing the additives were subjected to further mixing with the vulcanization system using a laboratory two-roll mill (Bridge). The composition of the rubber mixtures produced is illustrated in Table 1.

**Elemental analysis**

The contents of some metals in the samples were determined by the ICP-OES, Plasma Quant PQ 9000 Elite (Analytik, Jena, Germany). Standard solutions from Merck (1000 mg/L) were used for preparation of the calibration curves. Mineralization was carried out for 45 min at 240 °C under a pressure of 60 bar.

**SEM analysis**

The morphology of bio-filler samples was examined using a scanning electron microscope (SEM) (LEO 1530 Gemini, Zeiss, Jena, Germany). Additionally, the cross-sectional morphology of rubber composites was analyzed. Prior to the measurements, a carbon layer was deposited through the Cressington 208 HR system for preliminary processing.

**Contact angle measurements**

An optical goniometer (OCA 15EC, DataPhysics, Filderstadt, Germany) was used to determine the contact angle using the drop-based method. The contact angle was measured based on recorded images immediately after depositing a water drop (10 µL) on the surface of the vulcanizate.

**FTIR analysis**

Fourier-transform infrared spectroscopy (FTIR) was used to qualitatively assess additives obtained from alfalfa. The measurement range included radiation at a wavelength of between 4000 and 400 cm^−1^. The test parameters were a resolution of 8 cm^−1^ and 64 scans. The measurement was performed using a spectrophotometer (Thermoscientific Nicolet 6700, Thermo Fisher Scientific, Waltham, MA, USA) equipped with the Smart Orbit ATR attachment.

**Rheometric properties**

The rheometric properties were evaluated by measuring the torque as a function of time. The tests were conducted at a vulcanization temperature of 160 °C and a measurement duration of 15 min. Based on the rheometric parameters, all of the pre-prepared blends underwent vulcanization at a temperature of 160 °C for a duration determined by the measurements. The vulcanization process involved the application of pressure within the range of 100 to 150 bar.

**Differential scanning calorimetry**

The glass transition temperature and vulcanization kinetics of mixtures containing additives derived from alfalfa were determined using the differential scanning calorimetry (DSC) technique. Rubber samples (8–10 mg) were cooled to −150 °C and then heated to 250 °C at a heating rate of 10 °C/min. The measurements were carried out on a DSC1 calorimeter (DSC1, Mettler Toledo, Greifensee, Switzerland).

**Cross-linking density**

In order to determine the cross-linking density of vulcanizates, four samples of different shapes were cut from the rubber material, with weights ranging from 30 to 50 mg. These samples were placed in weighing bottles and immersed in toluene for 48 h to allow for equilibrium swelling. Subsequently, the samples were weighed and transferred to a dryer, where the solvent evaporated at 50 °C over the course of the next 48 h. After the drying process, the samples were reweighed to assess the changes in mass. The cross-linking density of the samples was calculated based on the Flory–Rehner theory [20].

The cross-linking density of NR composites was calculated as follows:

Q_w_—Equilibrium swelling value taking into account the NR composition:

(1) $$Q_{w}=\left(\frac{m_{sp}-m_{s}}{m_{s}}\right)\times \left(\frac{100+x}{100}\right)$$

m_sp_—mass of the swollen sample;

m_s_—mass of the dried sample;

x—the bioadditive content in the sample;

V_r_—the volume of the rubber fraction in the swollen gel, taking into account the value of the reduced equilibrium swelling by the content of bioadditives in the composition of the mixture [21]:

(2) $$V_{r}=\frac{1}{1+Q_{w}\frac{\rho_{r}}{\rho_{s}}}$$

$\rho_{r}$—rubber density [g/cm^3^];

$\rho_{s}$—solvent density [g/cm^3^];

γ_e_—concentration of effective chains (cross-linking density value):

(3) $$\gamma_{e}=\frac{\ln\left(1-V_{r}\right)+V_{r}+\mu V_{r}^{2}}{V_{0}\left(V_{r}^{\frac{1}{3}}-\frac{V_{r}}{2}\right)}$$

V_0_—molar volume of the solvent [mol/cm^3^];

µ—Huggin’s parameter (elastomer–solvent interaction) at 25 °C [22];

(4) $$\mu =\mu_{0}+\beta V_{r}$$

µ_0_—the parameter determines non-cross-linked polymer/solvent relations;

β—the parameter determines cross-linked polymer/solvent relations;

(µ_0_ = 0.478, β = 0.228).

**Mechanical properties**

The measurement of mechanical properties was performed using a universal testing apparatus (ZwickRoell). For each vulcanizate sample, W-3 dumbbell-shaped specimens were cut. The tensile strength test was performed at a speed of 500 mm/min, and the elongation was measured using an extensometer.

**Dynamic mechanical analysis**

In order to perform the measurement of dynamic mechanical analysis, circular samples were cut from the vulcanizates and subsequently subjected to investigation using the rotational rheometer (ARES-G2, TA Instruments, New Castle, DE, USA). Measurement took place at a temperature of 25 °C. An angular frequency of 10 rad/s was employed, and the strain was incrementally varied from 0.1% to 150%. To apply the necessary force on the samples, 25 mm parallel plates were utilized, and a force of 2N was uniformly applied. Afterwards, the Payne effect was calculated using Equation (5):

(5) $$∆G^{\prime }\;=\;G^{\prime }max\;-\;G^{\prime }min$$

*G*′max and *G*′min were taken from the range 0.1% to 100% of the strain since most of the results beyond those values were inaccurate.

**Barrier properties**

Barrier properties were evaluated based on the through-plane air permeability of vulcanizates using the manometric method. Numerically, they were defined by the gas transmission rate (GTR), which was calculated from the following Equation (6):

(6) $$GTR=\frac{V_{c}}{R\times T\times P_{u}\times A}\times \frac{dp}{dt}$$

*V_c_*—volume of the low-pressure chamber

*T*—measurement temperature

*P_u_*—gas pressure in the high-pressure chamber

*A*—measurement area, gas permeation area through the sample

*dp/dt*—pressure changes per unit of time

*R*—gas constant

**Thermogravimetric analysis**

The thermal stability of both NR elastomeric composites and plant raw material (alfalfa) was tested using thermogravimetric analysis (TGA). The measurements were made on a thermal analyzer (TGA/DSC1, Mettler Toledo) calibrated with indium and zinc standards. The test was performed in two stages. First, a sample of the material (approx. 10 mg) was heated at 25–600 °C, with argon flowing at 55 mL/min. The measurement was then continued up to 900 °C using air as the measurement gas. The heating rate was constant during the entire measurement and amounted to 20 °C/min.

**Ultraviolet aging**

NR composites were subjected to the following accelerated sharpening simulations: UV aging was performed using a UV 2000 camera from Atlas (USA). The vulcanizate samples were exposed in repeatable segments to the following conditions: daily segment (UV radiation intensity = 0.7 W/m^2^, temperature = 60 °C, duration = 8 h) and night segment (no UV radiation, temperature = 50 °C, duration = 4 h). The total aging time was 72 h.

**Thermo-oxidative aging**

Thermo-oxidative aging (TO) involved placing vulcanizate samples in a dryer (FD 56, Binder) with an air thermocirculator. The samples were exposed to elevated temperature (70 °C) for 14 days.

In order to determine the changes caused by aging factors acting on the samples, their mechanical properties were examined, and the color change was determined.

Based on the results of mechanical strength, the K factor was calculated according to Equation (7) [23]:

K = (TS × Eb)_after aging_/(TS × Eb)_before aging_ (7)

TS—tensile strength [MPa]; Eb—elongation at break [%].

**Color change**

The color change of vulcanizates caused by aging was examined spectrophotometrically using a spectrophotometer (CM-3600d, Konica Minolta, Chiyoda, Tokyo, Japan). The spectral measurement range was 360—740 nm. The marked color was subordinated to the color in the CIELAB color space. In the CIE uniform color space L*a*b*, the coordinates are L*—brightness coordinate; a*—red/green coordinate; b*—yellow/blue coordinate. The total color change of natural rubber vulcanizates was determined based on Equation (8) [24]:

(8) $$∆E=\sqrt{{\left(∆L\right)}^{2}+{\left(∆a\right)}^{2}+{\left(∆b\right)}^{2}}$$

## 3. Results and Discussion

### 3.1. Thermogravimetric Analysis of Plant Biomass

**Figure 1:**
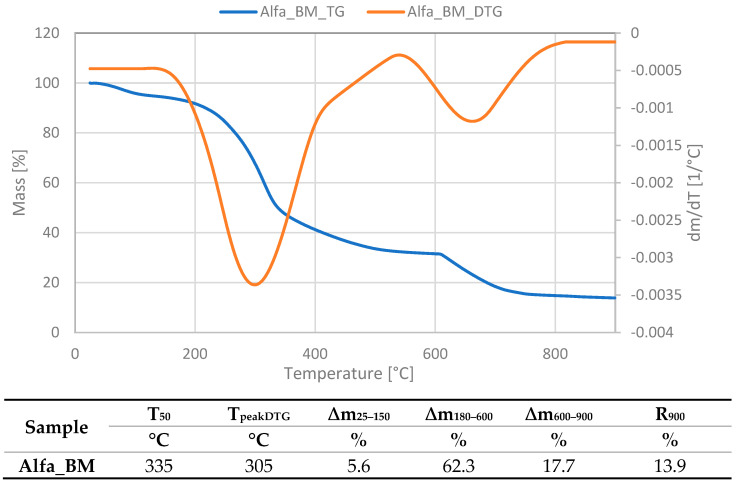
Thermogravimetric analysis of plant biomass.

The thermogravimetric (TG) analysis curve for plant biomass shown in Figure 1 illustrates the different stages of thermal degradation that correspond to different biomass components. Thermal decomposition of biomass from alfalfa took place in several stages, and the course of the TG and DTG curves is typical for this type of material.

The initial decrease in sample mass, observed at room temperature to about 150 °C, was relatively small (5.6%). It was related to the evaporation of moisture from the alfalfa sample. Although the material was pre-dried before measurement, it could contain some free water that is freely present in the intercellular spaces. In addition, there was also bound water in the lignocellulosic material, which refers to water that is an integral part of the plant structure and is retained in the material in a chemical or physical way. Then, the next stage was observed on the curves; this time, it was associated with a significant loss of mass, reaching 62.3%. It corresponded to the thermal decomposition of the lignocellulosic material, which for alfalfa was in the temperature range of 180–600 °C. Due to overlapping areas resulting from the simultaneous decomposition of alfalfa biomass components, there was one distinct peak on the DSC curve with a maximum decomposition rate of 305 °C. The temperature of 50% sample mass loss was similar and amounted to 335 °C; advanced decomposition occurred at this temperature. According to the literature, among the building blocks of plant biomass, hemicellulose is the least thermally stable and begins to decompose at approx. 150 °C and lasts up to 300 °C. In turn, cellulose decomposes in the range of 300–400 °C, which results in significant mass loss as a result of the decomposition of the cellulose structure into volatile compounds. Lignin decomposes over a wide temperature range (200–600 °C) due to its complex and non-homogeneous structure, in which aromatic groups occur [25,26]. Above 600 °C, the remaining biomass consists mainly of charcoal (carbon residue) and inorganic ash. The mass loss at this stage was relatively slow and represented the final decomposition of any remaining organic matter; it was 17.7% for the alfalfa studied. The mass remaining at the end of the TGA cycle, at 900 °C, represented the inorganic ash content of the biomass. The content of such compounds in alfalfa was 13.9%.

### 3.2. Elemental Analysis of Bioadditives

Understanding the qualitative and quantitative composition of powders used as bioadditives to elastomeric materials is extremely important from the point of view of their impact processing and utility properties. For this reason, an analysis of selected elements was conducted; the results are presented in Table 2.

Metals with variable valence, such as iron, copper, manganese, or chromium, can have a significant effect on the properties of elastomers. The content of such metals in elastomers can affect their mechanical, chemical, and thermal properties. These metals can occur in the form of salts, oxides, or as part of complex compounds. Metal compounds are often used in polymer processing technology. They are used as cross-linking agents, activators, and fillers [27,28,29].

Thermal treatment of biomass caused an increase in the concentration of metal content in the tested material. Among the elements examined, the highest contents of iron, aluminum, manganese, and zinc were noted in bioadditives obtained from alfalfa. Iron, copper, and zinc oxides are often used as substances supporting cross-linking reactions [30]; hence, their presence in biowaste samples may play an important role in the vulcanization process. In turn, heavy metals, such as copper (Cu), manganese (Mn), iron (Fe), and their salts, may act as catalysts for oxidation reactions in rubber. They may accelerate the aging process by generating free radicals or peroxides, which accelerate polymer degradation [31]. Plants also have the ability to accumulate slightly more harmful heavy metals, such as lead and cadmium, which were also detected in the tested materials derived from alfalfa. However, their content in the samples was very low.

### 3.3. FTIR Analysis of Bioadditives

**Figure 2:**
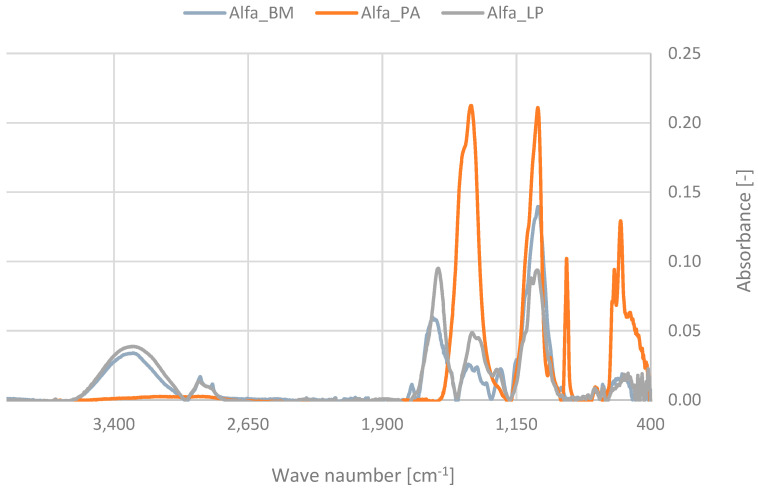
FTIR analysis of bioadditives.

IR spectroscopy was used to characterize bioadditives obtained from alfalfa. The spectra of the tested compounds are presented in Figure 2. The analysis provided information on the composition and structural properties of these materials.

Ash resulting from biomass combustion may contain various inorganic and organic components, and its chemical composition may vary depending on the type of plant subjected to high-temperature treatment and combustion conditions. In the spectrum recorded for alfalfa phyto-ash, we observed absorption bands typical of fly ash functional groups. The absorption bands in the regions 1000–1100 cm^−1^, 450–500 cm^−1^, 750–800 cm^−1^, and 950–1050 cm^−1^ can be assigned to the vibrations of Si-O-Si stretching, Si-O bending, and Al-O and Al-O-Si stretching, respectively [32,33]. Their presence may indicate the presence of compounds from the group of silicates and aluminosilicates in alfalfa-based phyto-ash samples. The biomass combustion process may cause the formation of carbonates, and the presence of these compounds in the ash sample confirms the presence of a band in the range of 1400–1500 cm^−1^ associated with C-O stretching vibrations [34]. The presence of carbon in the phyto-ash sample is also confirmed by the absorption band in the range of 1500–1600 cm^−1^, which is attributed to C=C stretching, indicating the content of aromatic compounds. In turn, the band in the range of 1600–1650 cm^−1^ indicates the presence of water or hydroxyl groups on the surface of the particles, as it refers to H-O-H bending [35]. Bands around 400–600 cm^−1^ are correlated with the occurrence of Fe-O stretching vibrations and confirm the presence of iron compounds in the biomaterial [36].

FTIR spectra of plant extracts typically show absorption bands corresponding to different functional groups, reflecting the complex mixture of bioactive compounds present. In the case of the spectrum recorded for the alfalfa extract, we can distinguish broad bands around 3200–3600 cm^−1^, which can be associated with O–H stretching. These are usually associated with alcohols, phenols, and water content in the plant extract [37]. The next intense absorption bands around 2850–2960 cm^−1^ and 1350–1470 cm^−1^ correspond to vibrations in the Alkyl groups range. These are C-H stretching and C-H bending, respectively. A very weak signal in the range of 1650–1750 cm^−1^ corresponds to C=O stretching. The presence of the carbonyl group indicates the presence of aldehydes, ketones, carboxylic acids, esters, and amides [38]. The band in the range of 1450–1600 cm^−1^, with a peak at 1580, is characteristic of C=C stretching vibrations in the aromatic ring. This type of structure can be attributed to the presence of aromatic compounds such as phenols and flavonoids [39]. The content of the extract sample of compounds from the group of esters and ethers is indicated by the presence of a band between the wavelength 1000 and 1300 cm^−1^, with peaks at 1080 and 1030 cm^−1^ corresponding to C-O stretching and C-O-C stretching vibrations.

Lignocellulosic materials, which include alfalfa plant biomass, are complex mixtures consisting mainly of cellulose, hemicellulose, and lignin. The FTIR spectra of these materials show characteristic absorption bands corresponding to the different functional groups present in these three main components. The presence of these compounds was confirmed based on the FTIR spectrum of the biomass. Broad bands around 3200–3600 cm^−1^ confirm the presence of hydroxyl groups in the tested material (O-H stretching), characteristic of the structure of cellulose and hemicellulose, as well as lignin, in which we distinguish phenolic hydroxyl groups [40]. Bands around 2800–3000 cm^−1^ indicate the presence of aliphatic C-H bonds in all three components.

The peak at 1740 cm^−1^ indicates the C=O stretching vibration, carbonyl group typically associated with acetyl groups, and uronic acid present in hemicellulose [41]. Bands around 1500–1600 cm^−1^ indicate the presence of aromatic structures because it is related to aromatic ring stretching (C=C) and is a characteristic absorption band for lignin. The next bands are related to C-O and C-C stretching vibrations and were recorded in the ranges 1000–1150 cm^−1^, 1160 cm^−1^ (cellulose and hemicellulose), and 1200–1300 cm^−1^ (phenols) [42]. These absorption bands provide a fingerprint for lignocellulosic materials, enabling the identification and characterization of the primary components and their structural features. The performed infrared spectroscopy analysis and interpretation of individual bands contributed to determining the composition of bioadditives isolated from alfalfa and helped to assess their impact on certain specific properties of elastomeric materials.

### 3.4. Morphology of Samples

**Figure 3:**
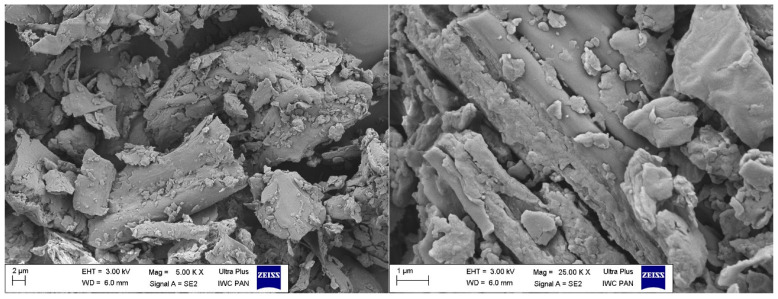
SEM images of biomass sample.

**Figure 4:**
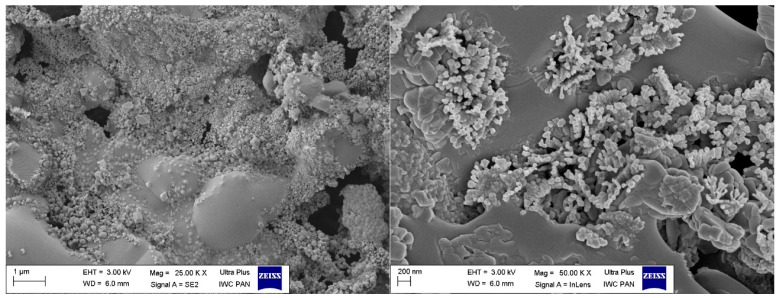
SEM images of the phyto-ash sample.

In order to determine the morphology of plant bio-filler samples in the form of both ground biomass and phyto-ashes produced during high-temperature processing of alfalfa, SEM images were prepared and studied (Figure 3 and Figure 4). The morphology of plant biomass showed a typical fibrous structure characteristic of plant materials, suitably crushed and used as typical bio-fillers of polymeric materials. The images presented showed both elongated larger forms of this material and smaller, crushed fragments of fibers, most probably crushed during the process of preparing the bioadditive (grinding dried alfalfa in a ball mill). The size and shape of the obtained particles, therefore, differed from each other, with their sizes ranging from about a few microns to about several hundred microns. Nevertheless, such typical fibrous characteristics of the biomass used may influence selected functional properties of the vulcanizates filled with them, such as increasing the barrier effect of gas permeability or mechanical reinforcement, although in this case, the appropriate dispersion of the material is also important, which in the case of fibrous material may be at a relatively low level.

In the case of morphological analysis of phyto-ashes, it should be taken into account that this material, which was confirmed in the elemental analysis, consists of, among others, metals with variable valences, such as iron, copper, manganese, aluminum, and chromium, and may also contain organic residues. The specific composition of phyto-ashes will affect its morphology after integration with the rubber matrix. In the case of the analyzed phyto-ash obtained as a result of high-temperature treatment of alfalfa, the bioadditives showed spherical shapes of particles with a relatively uniform distribution in the elastomeric matrix, even taking into account a certain tendency to combine into aggregates. Their size was definitely smaller compared to the fibrous biomass. Without a doubt, such structures of the bioadditive can improve the mechanical properties of the vulcanizates filled with them, both static and dynamic.

### 3.5. Rheometric Properties of Elastomer Mixtures

The characterization of rheometric properties of elastomer mixtures offers technological advantages, such as determining the optimal vulcanization time and assessing the flow of the composition. It also provides preliminary insights into the effect of fillers on the elastomer’s properties and their tendency to form a “structure” within the elastomer.

Trends in minimum torque reflect the viscosity and processability of the uncured rubber compound, while maximum torque correlates with the stiffness of the cured material. The recorded torque increase during rheometric tests is an indirect measure of the cross-link density, which in turn closely affects the mechanical characteristics of the composites.

Determination of the viscosity of elastomer mixtures was based on minimum torque measurements (Table 3). By comparing the obtained values with the reference sample, it can be concluded that systems containing bioadditives increase viscosity. This property is considered undesirable due to the increased difficulty of processing unvulcanized mixtures. Moreover, mixtures containing a smaller amount of bioadditive, regardless of its type, are characterized by much higher viscosity. However, as the amount of additive increases, this value gradually decreases. Importantly, this trend does not apply to samples containing extracts. Regardless of doubling the amount of lyophilisate added, the viscosity of these samples remains constant but still exceeds the reference value. The sample containing the highest amount of phyto-ash has the lowest viscosity value.

The increase in the torque value during mixture vulcanization can be closely related to the degree of cross-linking, as its increase correlates with the formation of cross-links between rubber macromolecules. It is worth noting that samples containing the highest amount of a specific bioadditive showed the greatest increase in cross-linking. However, it is worth adding that all tested samples with bioadditives showed a maximum torque value exceeding the value of the reference sample. An intriguing observation is that, as with the minimum torque, the maximum torque values for the freeze-dried samples showed negligible differences despite doubling the amount of extract used.

The scorch time is an important parameter in the processing of non-vulcanized elastomer mixtures, as its increase allows for the extension of technological process periods without material degradation. The inclusion of bioadditives had little effect on this parameter, and the lowest values were observed in samples containing phyto-ash as an additive. Moreover, regardless of the specific type of bioadditive used, the samples showed relatively similar scorch time values for each respective type of additive.

Similarly to the scorch time, the optimal curing time showed minimal differences when using different bioadditives. Moreover, it was very similar to the time recorded for the reference sample, which was approximately 2 min. Interestingly, however, samples containing phyto-ashes and extracts showed an extension of the optimal curing time with an increase in the amount of bioadditive. Conversely, in the case of biomass addition, a slight reduction in the optimal curing time was observed with increasing addition of biomass amounts.

### 3.6. Cross-Linking Density of Elastomeric Composites

**Figure 5:**
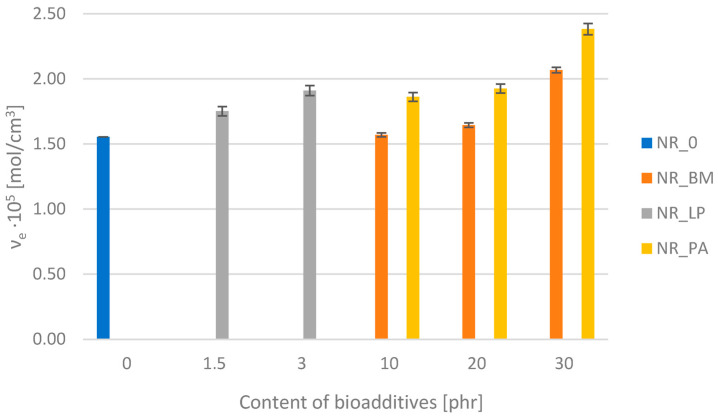
The value of cross-linking density of composites.

Cross-link density is a key indicator of the molecular structure of elastomers and has a significant impact on their mechanical and physical properties. The structures of bioadditives, such as phyto-ash, biomass, and freeze-dried extracts, affect the cross-link density of natural rubber vulcanizates through interaction with the rubber matrix and vulcanizing agents. These bioadditives can increase the cross-link density by providing active sites or reinforcing particles that promote the formation of additional cross-links, improving the mechanical and thermal properties of the material. The change in cross-link density can also reflect their influence on the cross-linking processes by modifying these bonds, which subsequently affect resistance to aging and degradation. The cross-link density of biocomposites was determined using the equilibrium swelling method. The analysis results are shown in Figure 5.

The obtained ν_e_ parameters indicate that each type of bioadditive used contributed to the increase in the cross-linking density of natural rubber compared to the reference sample. The greatest change in the ν_e_ value after filling was observed for elastomers containing phyto-ash. Ashes, especially those containing mineral components such as metal oxides (e.g., zinc oxide, magnesium oxide), can act as cross-linking activators [43,44]. Increased reactivity leads to the formation of a greater number of cross-links between polymer chains, which results in increased cross-linking density. The studies also showed an increased the ν_e_ value with increased phyto-ash content. A slight increase in cross-linking density was also observed for biocomposites filled with dried and ground alfalfa. Both ash particles and plant biomass can act as fillers in the elastomer matrix, influencing the distribution and structure of cross-linking. Fine particles of bio-fillers can facilitate the formation of cross-links around themselves, changing the local structure of the polymer network. As a result, they can affect the cross-link density. Their effect is dependent on their surface properties and interactions with rubber.

The addition of lyophilisates of alfalfa extracts had quite a significant effect on the results of the cross-link density of natural rubber. The analysis of equilibrium swelling showed that freeze-dried extracts actively affect the vulcanization reaction, increase the cross-link density, and promote the formation of more sulfide bonds during vulcanization.

### 3.7. Study of the Kinetics of Vulcanization and the Glass Transition Temperature of Biocomposites

The vulcanization of rubber mixtures is an exothermic process, which makes it identifiable as an exothermic peak on the DSC curve. Furthermore, by integrating this peak, the amount of heat released during vulcanization, i.e., the enthalpy of vulcanization (∆H), was determined. The temperatures at which the vulcanization process begins and ends (T_onset_ and T_endset_, respectively) were also determined. Additionally, based on the DSC analysis, the glass transition temperatures (Tg) of the tested rubber mixtures were determined. The above-mentioned parameters read from the DSC curve are listed in Table 4. According to the presented data, the vulcanization process for the reference sample, i.e., for unfilled natural rubber, proceeded in the temperature range of 155–216 °C, with an enthalpy of 13.3 J/g. Similar parameters for the course of vulcanization were obtained for the sample containing alfalfa biomass. In turn, the cross-linking process of compositions containing phyto-ash and lyophilisate of the extract began at a temperature about 30 °C lower, with a significantly higher energy effect. The obtained results indicate that compounds in phyto-ashes, as well as those occurring in plant extracts, can accelerate cross-linking reactions; what is more, they can increase their efficiency, leading to a more developed spatial structure, which was previously confirmed by the results of cross-linking density. The positive effect of the alfalfa extract on cross-linking reactions may result from the fact that plant extracts contain a relatively high content of flavonoids, phenolic acids, vitamins, and saponins, which can accelerate the vulcanization of sulfur in rubber, according to the literature [45]. In turn, the plant ash is rich in minerals, calcium, magnesium, zinc, and potassium compounds widely used in the rubber industry as substances supporting the vulcanization process [43,46,47].

No significant effect of the additives used on the glass transition temperature of natural rubber was observed. The Tg value for the unfilled NR sample was −62.7 °C, while compositions containing bioadditives were characterized by a slightly lower Tg, which was about −63 °C.

### 3.8. Dynamic–Mechanical Analysis of the Composites (Determination of the Payne Effect)

**Figure 6:**
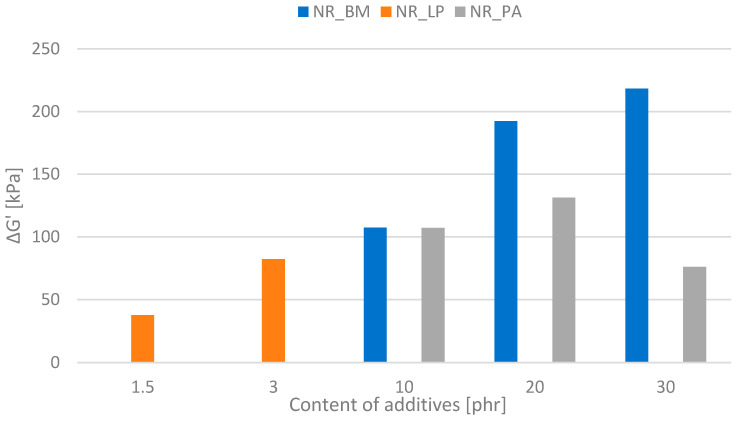
Payne effect values of the biocomposites.

The Payne effect (Δ*G*′) refers to the nonlinear behavior of elastomeric materials (rubbers) in the presence of large deformations, especially under small strains. The main manifestations of this effect are the decrease in the stiffness modulus (*G*′ modulus) with increasing strain in rubbers that contain fillers [48]. Fillers, especially those with a large surface area, tend to form agglomerates or networks in the elastomer structure. During small deformations, this network remains intact, which results in high material stiffness (high *G*′ modulus). However, under larger strains, this network partially disintegrates, which leads to a decrease in material stiffness, which is referred to as the Payne effect. The values of this parameter obtained for compositions containing alfalfa-based additives are presented in Figure 6.

The type and properties of the bioadditive had a significant effect on the size of the Payne effect. Among the additives tested, biomass had the greatest effect on the change in the stiffness of natural rubber subjected to dynamic shear stress. This means that the dried and ground alfalfa particles create an extensive network in the composite structure in the form of filler–polymer and filler–filler interactions. However, as the studies showed, these interactions were so weak that they were easily destroyed by the impact of the dividing deformation, which increased the intensity of the Payne effect. A larger amount of filler increased the Payne effect because more particles create agglomerates and increase the nonlinearity of the material’s response to deformation. The addition of phyto-ash also caused significant changes in the change of Δ*G*′ of natural rubber. At 10 phr content, the values for Δ*G*′ for composites containing PA and BM were similar, while at higher amounts of additive, NR_PA composites showed a decrease in the Payne effect value. Probably, phyto-ash particles interact more strongly with rubber than with each other. High concentrations of active fillers, which additionally and strongly interact with the elastomer matrix, may cause a decrease in the intensity of the Payne effect because the additives better stabilize the rubber structure and prevent the network from disintegrating during deformation. The presence of a greater number of polymer–phyto-ash interactions was also confirmed in studies determining the cross-linking density of biocomposites. Additionally, poorer dispersion of biomass in the rubber matrix may result in larger agglomerates that disintegrate more easily, thereby increasing the Payne effect in these vulcanizates. In the case of composites containing freeze-dried plant extracts, no significant effect on the Payne effect was noted, which results from the structure and nature of the additive used.

### 3.9. Mechanical Properties of the Biocomposites

**Figure 7:**
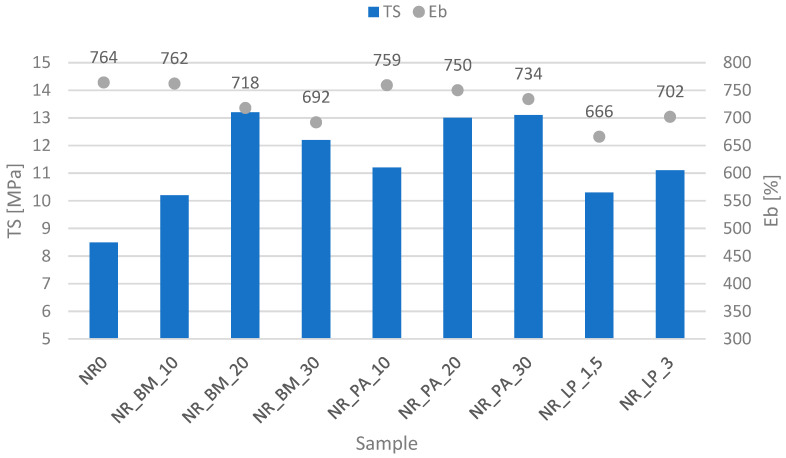
Tensile strength and elongation at break values of the vulcanizates.

Regardless of the type of bioadditive used, all composites showed a reduction in the Eb value compared to the reference sample (Figure 7). This is most likely due to the increased stiffness of the obtained materials after the incorporation of a more rigid solid phase in the form of filling. Generally, increasing the percentage of biomass and phyto-ashes resulted in a decrease in the elongation at break value. The opposite tendency was shown by vulcanizates with the addition of freeze-dried extracts, where doubling the share of the extract in the composite resulted in an increase in the Eb value. At the same time, these were the materials with the highest stiffness and, therefore, the lowest Eb values among all the systems produced.

In terms of tensile strength, each composite showed a higher value compared to the reference sample. The inclusion of bioadditives containing different components showed a positive effect on the increase in the mechanical strength of the final products, which is confirmed by the obtained values. The use of biomass and phyto-ashes resulted in a TS value of about 13 MPa. The increase in the amount of fillers influenced the improvement in strength, which confirms the previous observations from the studies of rheometric properties and cross-linking density, where a significant effect of the bioadditives used was observed both in the increase in rheometric moments and, understandably, the cross-linking density of the produced vulcanizates. Moreover, analyzing the SEM images from the previous paragraphs shows that such an effect could be expected, considering both the fibrous structures of the biomass and the evenly distributed phyto-ash particles. On the other hand, as can be expected, the use of freeze-dried materials did not have such an intense effect on the mechanical properties, although the obtained TS results were more than satisfactory.

The mechanical property profile of composites containing alfalfa-based additives is similar to that of other biocomposites of this type [49]. On the other hand, when compared to typical reinforcing fillers such as silica and carbon black, the mechanical properties are slightly inferior in vulcanizates filled with alfalfa additives [50]. However, it should be noted that in some industrial applications, mechanical strength in the range of 10–15 MPa is often entirely sufficient.

### 3.10. Thermo-Oxidative and UV Aging of the Biocomposites

In the context of samples subjected to combined thermal and UV aging, discrepancies are visible in the results obtained (Table 5). The rate of the aging reaction, which quantifies the extent of changes in material properties after aging, is approximately unity for both thermal and UV aging simulations for samples containing freeze-dried products. This suggests that the lyophilisate, as intended, exhibited consistent and stabilizing anti-aging activity in the context of both thermal and UV aging.

**Figure 8:**
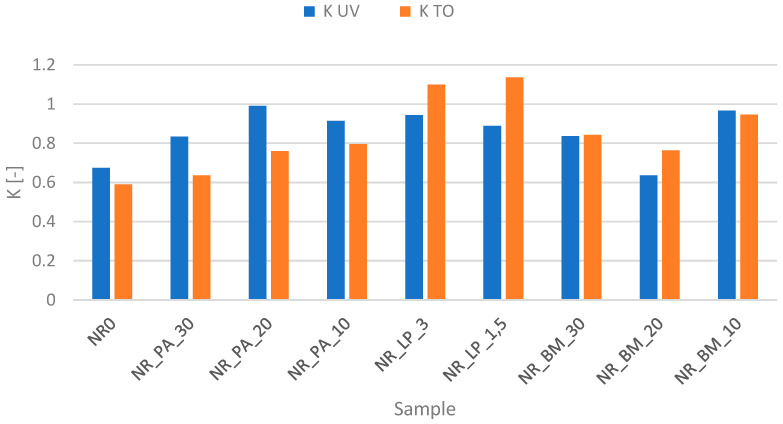
Aging factor values of biocomposites.

Moreover, increasing its share in the composite to 3 phr results in greater and more visible stabilization of the material (achieving an aging parameter value closer to 1). However, in the case of other categories of bioadditives, the results obtained introduce an element of ambiguity in this study of the properties of composites. In the case of samples filled with phyto-ash, these vulcanizates show slightly better stabilization in the context of thermal aging compared to UV. Moreover, increasing the percentage of filler in the samples results in deterioration of aging resistance. This may undoubtedly be related to the presence of metals (especially Mn, Fe, and Cu) with variable valence in these structures, which may contribute to catalyzing aging reactions [51,52]. In turn, when biomass is used, there is a noticeable tendency to deteriorate the properties of composites filled with higher fiber content; thus, the aging coefficient differs significantly from the value of 1 due to the higher share of alfalfa in the samples (Figure 8).

### 3.11. Analysis of the Color Change of the Vulcanizates

**Figure 9:**
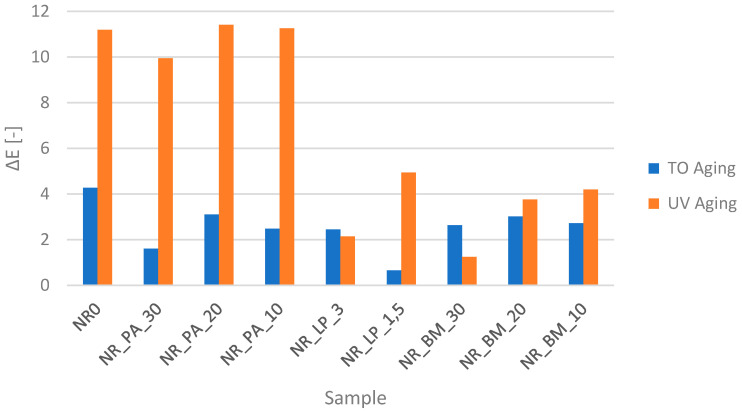
Change in the color of vulcanizates caused by aging processes.

Analysis of the color change of the composites determined after aging simulations allowed for a more in-depth look at the resistance to degrading factors of the manufactured materials filled with selected bioadditives (Figure 9). Based on the obtained results, it can be observed that, compared to the reference sample, the coefficient of total color change was consistently lower for all samples after thermo-oxidative (TO) aging, regardless of the type of bioadditive incorporated. The recorded changes, with values close to or below 3, indicate that the color alterations are not significant from an analytical standpoint. The sample with the lowest amount of freeze-dried extracts exhibited the lowest coefficient, while the NR_PA_20 sample demonstrated the highest value, although this was still lower than the reference value. Notably, a linear relationship was observed for samples containing lyophilisates, where an increased amount of the bioadditive correlated with a higher color change factor. In contrast, no discernible relationship was observed within the other sample groups.

The response to UV aging differs significantly from that of thermo-oxidative aging. Not all samples exhibit enhanced resistance to this specific aging condition compared to the reference sample. Notably, the NR_PA_10 and NR_PA_20 samples deviate from the trend and demonstrate a slightly higher coefficient of total color change than the reference sample. However, it is important to highlight the presence of linear correlations within each sample batch. Irrespective of the type of bioadditive used, an increase in its concentration in the blend results in a decrease or maintenance of the total color change coefficient at a similar level. Notably, the biomass blends exhibit the most pronounced improvement, while the phyto-ash samples show the least enhancement in terms of color stability.

### 3.12. Thermogravimetric Analysis of the Composites

**Figure 10:**
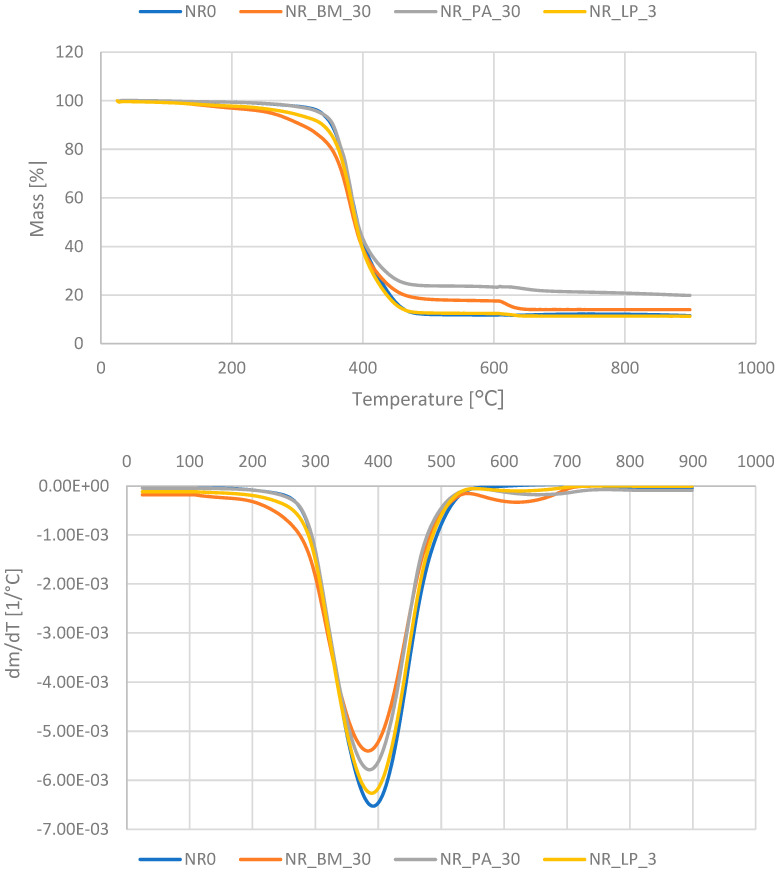
TG and DTG curves of the biocomposites.

The thermogravimetric analysis of natural rubber vulcanizates allowed us to determine the influence of the bioadditives obtained from alfalfa on the degradation and thermal stability of the rubber material. Moreover, this analysis allowed us to identify different degradation phases of the composite. TG and DTG curves of selected NR vulcanizates samples are illustrated in Figure 10, and characteristic parameters determined on the basis of their course are presented in Table 6.

In the lower temperature range (usually from room temperature to about 150–200 °C), there is usually a small loss of mass caused by the evaporation of water, moisture, and volatile organic substances. Among the tested samples, only the one containing biomass showed a small loss of mass in the discussed range, related to the removal of water in alfalfa fibers. The remaining vulcanizates maintained thermal stability up to 200 °C.

At higher temperatures, from about 200 °C to 600 °C, the main thermal degradation process of the rubber composite occurred, including the decomposition of natural rubber and organic fillers. At this point, a large loss of material mass is observed. In the case of the reference sample and the composite containing the freeze-dried material, the mass loss up to 600 °C (∆m_25–600_) was about 88% and corresponded to the rubber content in the material. In turn, the remaining composites showed a smaller percentage loss of mass due to the significant addition of bio-fillers. Because phyto-ashes remain stable up to 600 °C for NR_PA composites, it was much smaller. In the NR_BM composite, the contained biomass partially decomposed, contributing to the increase in the value of the loss ∆m_25–600_.

In the final phase of TGA measurement above 600 °C, conducted in oxidizing conditions, the mass loss of the rubber composite is related to the combustion of matter formed after the decomposition of organic components. The largest mass loss in this range (∆m_600–900_) was noted for the composite containing biomasses (3.6), corresponding to the degradation of the carbon content of alfalfa fibers. In the remaining filled composites, slightly lower values of the sample mass loss were observed. The solid residue of the sample at 900 °C (R_900_) refers to the content of mineral compounds in the material. From this level, the content of inorganic substances (ash residue) is determined. For the reference sample, this value was 11.5%; its composition usually consists of ZnO, acting as an activator, and impurities present in natural rubber, the so-called non-rubber compounds. A similar R_900_ value was shown by the sample containing the lyophilizate. The highest value of solid residue after thermal analysis (ca. 21%) was characterized by the vulcanizate containing PA, which additionally enriched the solid residue with plant phyto-ash containing thermally stable mineral substances.

### 3.13. Barrier Properties of the Vulcanizates

**Figure 11:**
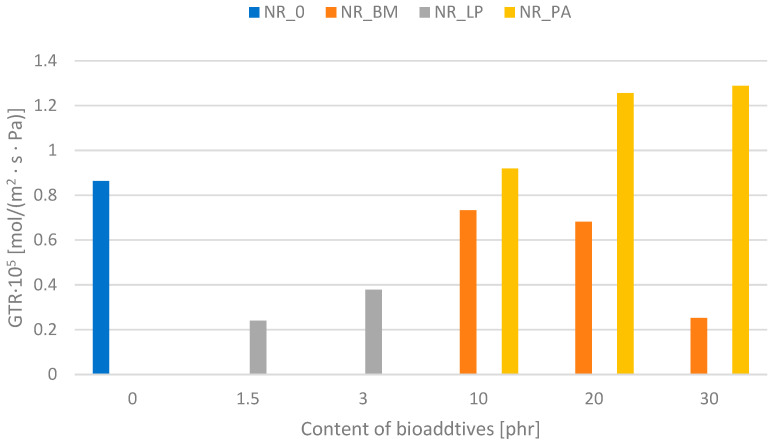
Gas transmission rate (GTA) characteristic of the NR composites.

The analysis of barrier properties based on the change in the gas transmission rate (GTR) showed that the addition of fillers in the form of biomass significantly affects the change in this parameter (Figure 11). Moreover, a clear correlation is noticeable, which indicates that with the increase in the filler content, the barrier effect of the vulcanizates produced for the penetrating gas increases. This is undoubtedly related to the addition of the fibrous phase of the solids, which constitutes a blockade for the gas penetrating through the sample [53]. It is also worth adding that in each case, the addition of biomass improves the barrier properties relative to the reference samples. A completely opposite trend was observed for composites filled with phyto-ash. Ashes, as a residue after high-temperature processing, do not constitute any barrier for gas streams penetrating through the sample. This may be due to the fact that the filler particles improve the gas flowing through the vulcanizate due to their shape and even distribution in the composite.

Without a doubt, the lyophilisates used had a very large impact on the barrier effect of the produced vulcanizates. Freeze-dried natural extracts can modify the permeability of rubber vulcanizates by changing the physical and chemical interactions in the material, creating complex pathways that impede gas flow and improving the overall barrier properties of the rubber mixture. This may be the result of several different mechanisms. First, when lyophilized extracts are incorporated into rubber, they can affect the microstructure of the vulcanizate. If the extracts are large or form physical networks in the rubber matrix, they can block the pathways through which gas molecules move, effectively increasing the material’s resistance to gas permeation. Second, natural extracts can participate in or promote additional cross-linking within the vulcanizate. Enhanced cross-linking can create a tighter network structure, reducing gas movement in the matrix. Third, the chemical properties of natural extracts may also play a role; for example, they may form hydrogen bonds or other intermolecular interactions with the rubber, affecting the way gases permeate the material.

### 3.14. Determination of Hydrophobic Properties of Biocomposites

The provided outcomes indicate that the reference sample manifests characteristics aligned with hydrophobic and non-polar tendencies, with the measured contact angle being the highest and amounting to 103.56° (Table 7). Analogous trends are discernible in the case of samples augmented with biomass additives and lyophilized materials, with disparities in their values being virtually negligible, reaching CA values of about 100°. Conversely, contrasting results are observed for samples containing phyto-ashes. In this instance, an increased quantity of added phyto-ashes correlates with a diminishing trend in the contact angle value. Phyto-ashes, particularly those sourced from natural sources, can contain organic compounds, minerals, and residues from the plant material. When these ashes are added to a natural rubber blend, they may introduce additional polar and hydrophilic functional groups to the composite’s surface. This can effectively enhance the affinity of the composite’s surface for polar substances like water, reducing the contact angle.

## 4. Conclusions

The presented work has shown that plant materials, especially alfalfa, can be processed to obtain various forms of active substances of natural origin, which can act as bioadditives in rubber composites. Depending on the processing method, materials with different structures, morphologies, and properties were obtained. Each of the obtained forms of bioadditives obtained from alfalfa (crushed biomass, lyophilisates from the extract, and phyto-ash) was characterized by other specific parameters, simultaneously influencing the modification of selected processing and functional properties of polymer composites filled with them.

Crushed alfalfa biomass allowed us to obtain a filler with varied morphology, where fibrous particles dominated. This type of additive to NR proved to be a semi-reinforcing filler, positively influencing processing processes and spatial structure of composites, and thus contributed to increasing mechanical strength. Moreover, fibrous elements increase resistance to gas penetration, creating a physical barrier that hinders diffusion through the material. In turn, phyto-ashes rich in mineral substances can be a natural source of co-agents supporting the processes of sulfur vulcanization of rubber, actively influencing the efficiency of the cross-linking reaction. As a result, the addition of phyto-ashes to rubber contributed to the improvement of functional properties, such as tensile strength, reduced gas permeability, and increased thermal stability. In turn, water–alcohol extraction of alfalfa herb allowed us to obtain active compounds that significantly contributed to the stabilization of the elastomeric material in terms of resistance to thermo-oxidative and ultraviolet aging.

## Figures and Tables

**Table 1:** Formulation of polymer mixtures.

	NR	Biomass	Phyto-Ashes	Lyophilisate	ZnO	MBT	S	SA
	phr	phr	phr	phr	phr	phr	phr	phr
**NR0**	100				5	2	2	1
**NR_BM_10**		10						
**NR_BM_20**		20						
**NR_BM_30**		30						
**NR_PA_10**			10					
**NR_PA_20**			20					
**NR_PA_30**			30					
**NR_LP_1.5**				1.5				
**NR_LP_3**				3				

phr—parts per hundred of rubber.

**Table 2:** Elemental analysis of bioadditives.

	Zn	Pb	Ni	Cu	Fe	Sr	Cr	Al	Mn
g/kg									
**Alfa_BM**	0.0023	0.00004	0.0002	0.0011	0.0216	0.0074	0.0002	0.0323	0.0033
**Alfa_PA**	0.0227	0.00011	0.0013	0.0082	0.2131	0.0753	0.0010	0.2114	0.0346

**Table 3:** Rheometric characteristics of NR composites filled with bioadditives.

Sample	M_min_	M_max_	TC_5_	TC_90_
	dNm	dNm	Min	Min
**NR0**	0.43	4.87	0.63	2.21
**NR_BM_10**	0.81	5.16	0.58	2.13
**NR_BM_20**	0.71	6.20	0.56	2.05
**NR_BM_30**	0.73	7.25	0.52	1.91
**NR_PA_10**	0.82	5.97	0.44	1.85
**NR_PA_20**	0.78	6.62	0.44	1.90
**NR_PA_30**	0.56	6.96	0.45	2.15
**NR_LP_1.5**	0.69	5.41	0.50	1.85
**NR_LP_3**	0.69	5.44	0.49	1.90

**Table 4:** DSC analysis of selected rubber mixes.

Sample	Content of Filler	T_onset_	T_endset_	∆H	Tg
	[phr]	[°C]	[°C]	[J/g]	[°C]
**NR0**	0	155	216	13.31	−62.7
**NR_BM_30**	30	154	213	17.74	−63.4
**NR_PA_30**	30	124	212	38.94	−63.0
**NR_LP_3**	3	121	223	30.76	−63.0

T_onset_—Initial temperature of vulcanization; T_endset_—Final vulcanization temperature; ∆H—Enthalpy of the vulcanization process; Tg—Glass transition temperature.

**Table 5:** Changes in the mechanical properties of vulcanizates under the influence of thermo-oxidative and UV aging.

Sample	TS	Eb	TS	Eb	TS	Eb
	MPa	%	MPa	%	MPa	%
	Before Aging		After UV Aging		After TO Aging	
**NR0**	8.5	764	5.8	747	5.3	721
**NR_BM_10**	10.2	762	9.9	757	9.9	744
**NR_BM_20**	13.2	718	8.9	677	10.1	712
**NR_BM_30**	12.2	692	10.5	672	10.4	684
**NR_PA_10**	11.2	759	10.4	747	9.23	733
**NR_PA_20**	13.0	750	13.2	732	10.2	726
**NR_PA_30**	13.1	734	11.1	722	8.7	705
**NR_LP_1.5**	10.3	666	9.27	658	11.9	655
**NR_LP_3**	11.1	702	10.5	700	11.7	732

**Table 6:** Thermogravimetric analysis of NR biocomposites.

Sample	T_50_	∆m_25–600_	∆m_600–900_	R_900_
	°C	%	%	%
**NR0**	391	88.2	0.2	11.5
**NR_BM_30**	387	82.4	3.6	14.0
**NR_PA_30**	397	76.7	2.4	20.9
**NR_LP_3**	389	87.5	1.1	11.3

**Table 7:** Contact angle values measured for NR vulcanizates.

Sample	Water
	CA [°]
**NR0**	103.56
**NR_BM_10**	100.00
**NR_BM_20**	91.66
**NR_BM_30**	99.64
**NR_PA_10**	97.38
**NR_PA_20**	86.44
**NR_PA_30**	75.49
**NR_LP_1.5**	101.96
**NR_LP_3**	100.15

## Data Availability

The raw data supporting the conclusions of this article will be made available by the authors on request.
